# A Novel Allele Encoding 7-Hydroxymethyl Chlorophyll a Reductase Confers Bacterial Blight Resistance in Rice

**DOI:** 10.3390/ijms22147585

**Published:** 2021-07-15

**Authors:** Marie Gorette Kampire, Ringki Kuinamei Sanglou, Huimei Wang, Bello Babatunde Kazeem, Jian-li Wu, Xiaobo Zhang

**Affiliations:** 1 State Key Laboratory of Rice Biology, China National Rice Research Institute, Hangzhou 310006, China; kamgorette@gmail.com (M.G.K.); ringki007@gmail.com (R.K.S.); wanghuimei@caas.cn (H.W.); 2 Lianyungang Academy of Agricultural Sciences, Lianyungang 222006, China; tunlapa2k13@gmail.com

**Keywords:** rice, bacterial blight, defense response, *OsHCAR*

## Abstract

Rice spotted leaf mutants are helpful to investigate programmed cell death (PCD) and defense response pathways in plants. Using a map-based cloning strategy, we characterized novel rice spotted leaf mutation *spl^HM143^* that encodes a 7-hydroxymethyl chlorophyll a reductase (*OsHCAR*). The wild-type (WT) allele could rescue the mutant phenotype, as evidenced by complementation analysis. *OsHCAR* was constitutively expressed at all rice tissues tested and its expression products localized to chloroplasts. The mutant exhibited PCD and leaf senescence with increased H_2_O_2_ (hydrogen peroxide) accumulation, increased of ROS (reactive oxygen species) scavenging enzymes activities and TUNEL (terminal deoxyribonucleotidyl transferase-mediated dUTP nick-end labeling) -positive nuclei, upregulation of PCD related genes, decreased chlorophyll (Chl) contents, downregulation of photosynthesis-related genes, and upregulation of senescence-associated genes. Besides, the mutant exhibited enhanced bacterial blight resistance with significant upregulation of defense response genes. Knockout lines of *OsHCAR* exhibited spotted leaf phenotype, cell death, leaf senescence, and showed increased resistance to the bacterial pathogen *Xanthomonas oryzae* pv. *oryzae* *(Xoo)* coupled with upregulation of five pathogenesis-related marker genes. The overexpression of *OsHCAR* resulted in increased susceptibility to *Xoo* with decreased expression of pathogenesis-related marker genes. Altogether, our findings revealed that *OsHCAR* is involved in regulating cell death and defense response against bacterial blight pathogen in rice.

## 1. Introduction

Rice bacterial blight is a deadly bacterial disease which is among the most destructive for cultivated rice (*Oryza sativa*). Spotted leaf mutants of rice known to provide extensive resistance to different bacterial pathogens serve as a great material to elucidate disease resistance mechanisms in rice. Understanding the functions of disease-resistant genes is essential for unraveling the molecular mechanisms of plant capacity to withstand pathogenic bacteria and improve plant adaptability to stress through genetic programming [1]. In rice, spotted leaf mutants are known to develop necrotic lesions spontaneously without any environmental stress intervention. The developed necrotic lesions are mainly observed on the leaf blade or leaf sheath and are of different sizes (2–10 µm) and multiple colors such as brown, reddish-brown, dark brown, orange, and white [2,3]. Spotted leaf mutants are also termed lesion mimic mutants (LMMs) because they display either structural or uncontrolled cell death initiation similar to the hypersensitive response (HR) induced by pathogen infection [4]. HR is associated with typical physiological processes that comprise structural signals such as the initiation of pathogenesis-related (PR) genes, the outburst of ROS, the aggregation of antimicrobial compounds, and the generation of free radicals [5,6,7].

Genetic studies have stated that spotted leaf mutants are commonly governed by single recessive genes [8,9], single semi-dominant nuclear genes [10] or single dominant genes [11]. In recent years, many spotted leaf mutant genes have been cloned. They encode several proteins with distinct functions and signaling pathways associated with defense response and HR cell death in rice [12]. These proteins include ATP-citrate lyases (ACL) [13], spastin protein [14], coproporphyrinogen III oxidase [15], mitogen-activated protein kinase kinase kinase (MAPKKK1) [16], eEF1A-like protein [7] tetratricopeptide repeats (TPRs)-containing protein [17], and AAA-type ATPase [18]. These findings specify that several proteins are important regulators of HR cell death and immunity response [13].

Most spotted leaf mutants show improved defenses against bacterial and fungal pathogens. For example, rice *spl40* mutant displayed increased resistance to bacterial blight pathogen *Xoo* [19], whereas *Spl12*, *spl13*, *spl14* and *Spl15* show enhanced resistance to not only *Magnaporthe oryzae* but also *X. campestris* pv. *oryzae* [20], and *spl33* exhibited improved resistance to *Magnaporthe oryzae* and *Xoo* [7]. These data imply that spotted leaf genes participate in the regulation of the plant defense response. Certain gene products such as phosphates, G-proteins, and protein kinase, other signal molecules such as lipid peroxides, ROS, salicylic acid (SA), and jasmonic acid (JA) are produced during the defense response. In fact, at the beginning of defense response, many biochemical pathways within the responding cells are immediately activated, followed by the induction of defense-related genes, including genes encoded by chitinase, phenylalanine ammonia-lyase (PAL), and, chalcone synthase (CHS) plus protectant genes including genes encoding glutathione S-transferase (GST), glutathione peroxidase (GP) and peroxidases for activating cell protection mechanisms [21]. During the last decade, several LMM have been isolated and cloned. It is now recognized that these mutants are valuable materials for studying various features of PCD and the disease resistance mechanisms of rice [4].

7-hydroxymethyl chlorophyll a reductase (HCAR) is an enzyme of Chl cycle that accelerates the modification of 7-hydroxymethyl Chl a (7-HMC a) to Chl a [22]. In rice and *Arabidopsis*, HCAR is considered a component of Chl catabolic enzyme (CCE). It interferes with RCCR, SGR, NOL, and NYC1 in yeast two-hybrid experiments, suggesting its prominent role in Chl degradation [23,24]. A recent study demonstrated that HCAR acts as one of the suppressing factors for Chl b turnover when Chl b is overproduced. Analysis performed on Chl-b-overproducing plants and the lines that overexpress HCAR showed that the Chl b level was decreased. In contrast, the Chl a/b ratio was elevated from about 0.8–1 to 1.6–2, implying that HCAR activity was not enough to convert HMChl a reduction in Chl-b-overproducing plants [25]. In rice, HCAR has been reported to play a crucial role in saving plants from high light-induced cell injury by blocking the aggregation of 7- HMC a and Pheo a in evolving and mature leaves during plant development [24]. However, an association between disease resistance and HCAR has not been reported in plants so far. 

Previously, a spotted leaf mutant obtained by the EMS (Ethyl methane sulfonate)-induced IR64 mutant bank (*HM143*) showed necrotic lesions on the leaf blades and exhibited broad spectrum resistance to different bacterial blight pathogens [9]. To confirm whether the mutation in *HM143* was associated with the spotted leaf phenotype and bacterial resistance we carried out functional analysis of this gene which would deepen our understanding of the molecular mechanisms underlying disease resistance in rice. Here, we isolated *spl^HM143^*, which encodes 7-hydroxymethyl chlorophyll a reductase, hereafter (*OsHCAR*). A single base exchange in the mutant allele leading to an mRNA splicing is responsible for the mutant phenotype indicated by complementation test. The *OsHCAR* gene is constitutively expressed in all tissues examined and *OsHCAR* localizes to chloroplasts. The mutant displayed cell death, leaf senescence and increased defense response. The knockout lines of *OsHCAR* exhibited similar spotted leaf phenotype, leaf senescence, cell death and strengthened disease resistance. Our results demonstrated that dysfunction of *OsHCAR* could activate the defense pathway and lead to enhanced defense response in rice.

## 2. Results

### 2.1. PCD Is Activated in HM143

**Figure 1:**
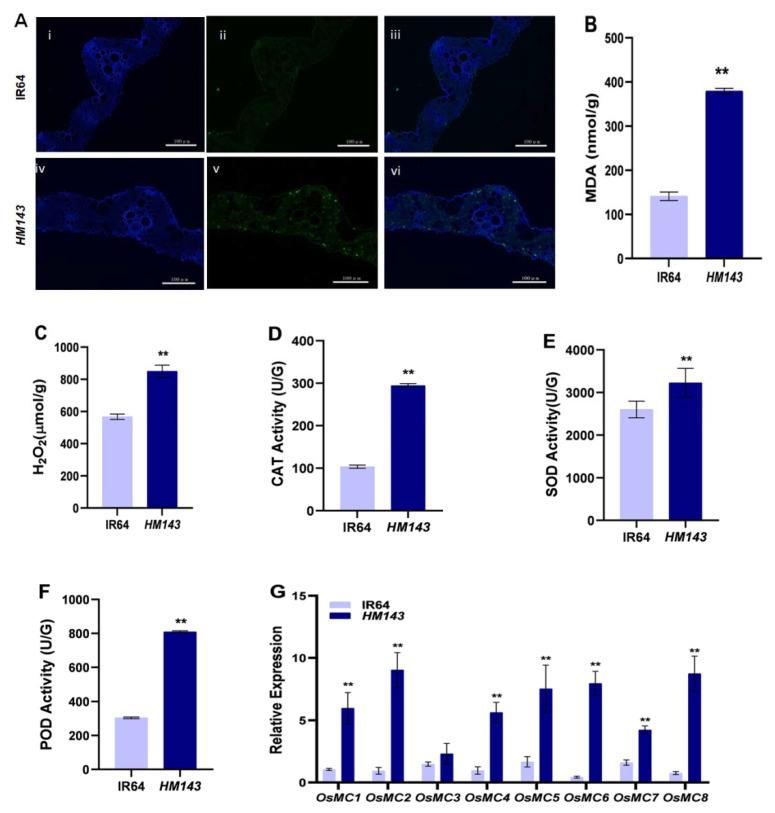
Analysis of cell death and ROS accumulation in IR64 and *HM143* at tillering stage. (**A**) TUNEL assay. Blue signal represents 4′, 6-diamino-phenylindole (DAPI) staining; green color represents positive result. (i) and (iv) are DAPI staining; (ii) and (v) are TUNEL signal; (iii) and (vi) are merged images of (i/iv) andrespectively, Bar = 100 µm; (**B**) Malonaldehyde (MDA) contents; (**C**) H_2_O_2_ contents; (**D**–**F**) CAT, SOD and POD enzyme activities (**G**) Expression analysis of *OsMCs* genes. Data are means ± SD of three biological replicates (Student’s *t*-test: * ** *p* < 0.01).

A previous study denoted that cell death occurred at every site of necrotic lesions, followed by the cumulation of hydrogen peroxide in *HM143* [9]. To further confirm the presence of cell death in *HM143*, we performed TUNEL analysis to detect DNA fragmentation, which indicates cell death. We observed few positive TUNEL nuclei in the WT, whereas many TUNEL nuclei were positive in *HM143* (Figure 1A). We also measured the levels of malonaldehyde (MDA) and H_2_O_2_. Our results revealed that the contents of both MDA and H_2_O_2_ were significantly increased in *HM143* compared with WT (Figure 1B,C), indicating that cell membrane damage caused cell death in *HM143*.

To verify whether anti-oxidative systems, consisting of catalase (CAT), superoxide dismutase (SOD), and peroxidase (POD) enzymes, were elevated to balance ROS production, we determined the activities SOD, POD and CAT between mutant and WT. We found out that the all the three enzyme activities were significantly higher in *HM143* when compared with WT (Figure 1D–F). Our results imply that increased activities of ROS scavenging enzymes could not lower the elevated H_2_O_2_ accumulation. Lastly, we performed the expression analysis of several metacaspase (MC) genes, which are essential controllers of PCD [26]. The results revealed that seven MC genes, including *OsMC1*, *OsMC2, OsMC4, OsMC5, OsMC6, OsMC7*, and *OsMC8*, were highly expressed in *HM143* compared to WT, but no remarkable difference was detected in the expression pattern of 1 MC gene, i.e., *OsMC3* between *HM143* and WT (Figure 1G). Our results demonstrated that *HM143* triggers the PCD pathway, which led to the formation of HR-like necrotic lesions.

### 2.2. HM143 Is Deficient in Chlorophyll Metabolism and Photosynthesis

**Figure 2:**
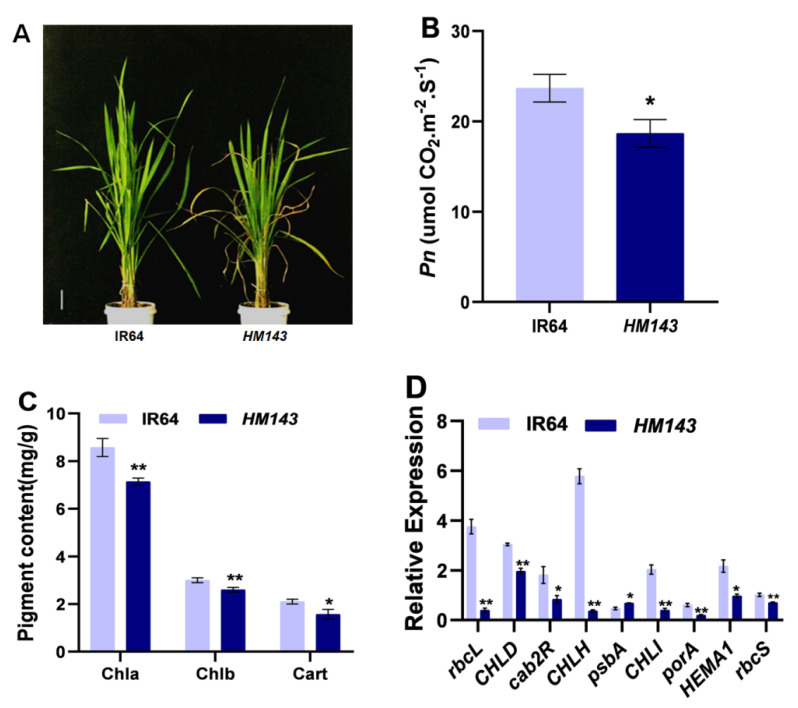
Analysis of leaf senescence in IR64 and *HM143* at the tillering stage. (**A**) Phenotypes of IR64 and *HM143* at the tillering stage, bar = 17 cm; (**B**) Net Photosynthetic rate (*Pn*); (**C**) Pigment content of IR46 and *HM143*; (**D**) Expression levels of photosynthetic related genes. Data are means ± SD of three biological replicates (Student’s *t*-test: * *p* < 0.05; ** *p* < 0.01).

Photosynthetic rate (Pn) and Chl content are the two significant characteristics of leaf senescence in plants [27]. To examine early leaf senescence, we measured the levels of Chl contents in mutant and WT plants at the tillering stage (Figure 2A). We found that in *HM143,* Chl a, Chl b, and carotenoid (Cart) levels were notably lower than WT (Figure 2C), suggesting that necrotic lesions’ formation in *HM143* might have caused the reduction of Chl contents. We also observed that the net photosynthetic rate was significantly decreased in the *HM143* plants as opposed to WT (Figure 2B). We also evaluated the expression levels of eight photosynthesis-related marker genes (*rbcL*, *CHLD*, *cab2R*, *CHLH*, *psbA*, *CHLI*, *porA*, *HEMA1*, *rbcS*) by qRT-PCR (Quantitative Real-Time Polymerase Chain Reaction) in the mutant and WT. All the photosynthesis-related genes tested were remarkably downregulated in the mutant compared to the WT (Figure 2D), providing a shred of molecular evidence for leaf senescence in the mutant. Our results imply that *HM143* is deficient in Chlmetabolism and photosynthesis.

### 2.3. spl^HM143^ Encodes 7-Hydroxymethyl Chlorophyll a Reductase (OsHCAR)

**Figure 3:**
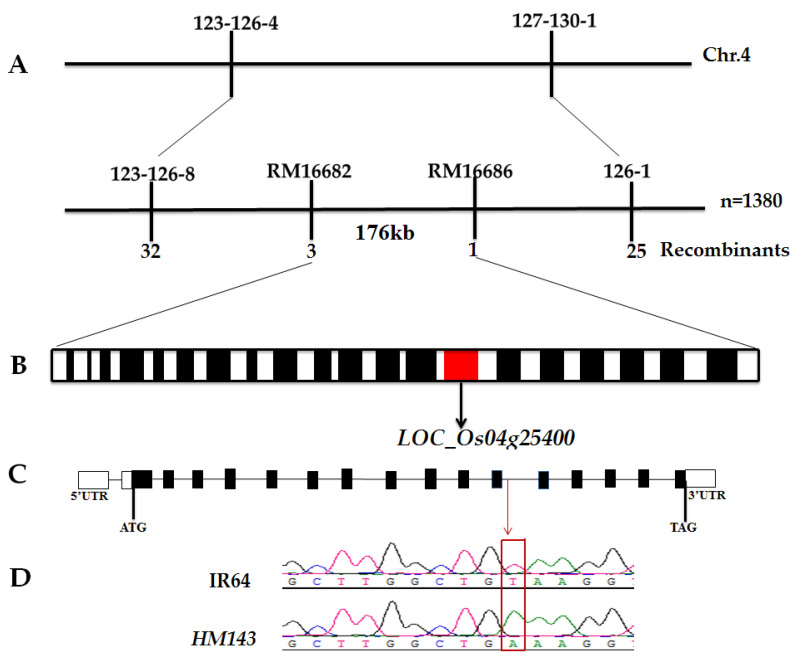
Map based cloning of *OsHCAR*. (**A**) *OsHCAR* gene location on chromosome 4 between markers RM16682 and RM16886. (**B**) 20 ORFs were located in 176kb region, red box represents *LOC_Os04g25400*, and black boxes represent other ORFs. (**C**) Gene structure of *LOC_Os04g25400*, white boxes represent 5′UTR and 3′UTR, respectively, while black boxes represent coding exons, lines represent introns. (**D**) Sequence analysis of the T to A point mutation in WT and *HM143* on the second nucleotide of 12^th^ intron. Note: The numbers 123-126-4,127-130-1,123-126-8,126-1 indicate insertion/deletion markers.

In a previous study, the single recessive gene *spl^HM143^* was preliminarily mapped to chromosome 4 [9]. A sum of 1380 F_2_ individuals mutant-type obtained from the cross *HM143*/Moroberekan was selected for genotyping to fine map the locus. The candidate gene was located to a 176Kb region between RM16682 and RM16686 markers (Figure 3A). Elicited from the Rice Genome Annotation Project database (http://rice.plantbiology.msu.edu/, accessed on 23 June 2021), the fine mapped region contains 20 putative open reading frames (ORFs) (Figure 3B). Sequencing and comparing all 20 ORFs from WT and *HM143* indicated that the 14th ORF (*LOC_Os04g25400*) had one nucleotide exchange from T to A at position 3760 in its 12th intron splicing site (Figure 3D) resulting in mRNA splicing, thus considering *LOC_Os04g25400* as the most likely candidate gene. The length of *LOC_Os04g25400* is 5273 bp with 16 exons and 16 introns (Figure 3C). *LOC_Os04g25400* is predicted to encode 7-hydroxymethyl chlorophyll a reductase (HCAR), hence named *OsHCAR*.

**Figure 4:**
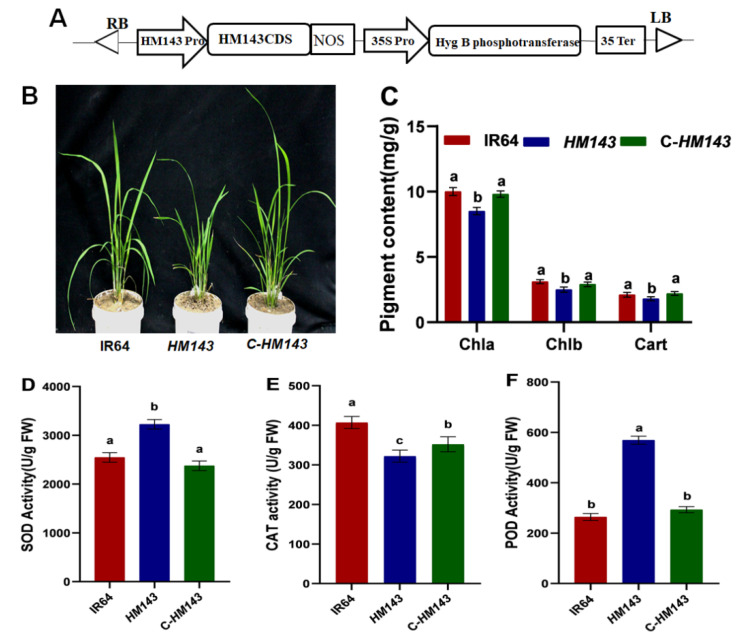
Genetic complementation of the mutant. (**A**) Complementation construct. (**B**) Phenotypes of IR64, *HM143* and complemented transgenic line (*C-HM134*). (**C**) Pigment contents in IR64, *HM134* and *C-HM143* at heading stage. (**D**–**F**) Enzyme activities of SOD, CAT and POD in IR64, *HM143* and C-*HM143* at heading stage. Data are means±SD of three biological replicates. Different letters indicate significant differences according to One-way ANOVA and Duncan’s test (*p* ≤ 0.05).

To confirm the function of the candidate gene, we performed *Agrobacterium tumefaciens*-mediated transformation of the wild *OsHCAR* allele in the mutant calli (Figure 4A). Eight T_0_ plants were regenerated; six were positive transformants and displayed the same regular green leaf color as the WT(Figure 4B); while the other two showed the same lesion mimic phenotype as the mutant (Supplementary Figure S1A,B). We also measured the total Chl content in IR64, *HM143*, and C-HM143. Our results showed that the total Chl level in C-HM143 recovered to the WT levels while it significantly lowered in *HM143* (Supplementary Figure S1C). Additionally, when we measured the content of Chl, the mutant showed decreased levels of Chl a, b, and Cart compared with WT: these parameters rescued WT levels in complementary plants (Figure 4C). Furthermore, we also measured the enzyme activities of POD, SOD and, CAT and our results showed that POD and SOD activities in the complementary plants fully recovered to the WT level (Figure 4D,F). In contrast, the activity of CAT was decreased compared to WT but higher than that of *HM143* (Figure 4E). Joined together, these findings illustrated that *LOC_Os04g25400* is the candidate gene that regulates the mutant phenotype.

### 2.4. Overexpression of OsHCAR Enhances Susceptibility to Xoo

**Figure 5:**
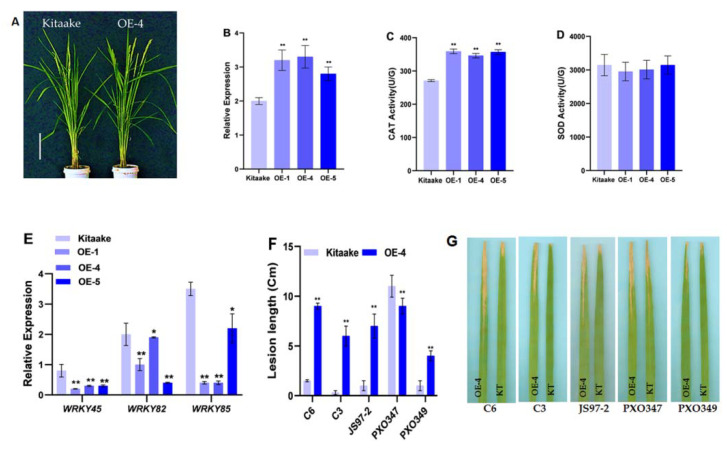
Overexpression analysis of *OsHCAR* in Kitaake background. (**A**) Phenotypes of Kitaake and T_1_ Overexpression line (OE-4). (**B**) Relative expression of OsHCAR in Kitaake and different OE lines. (**C**,**D**) Enzyme activities of CAT and SOD (**E**) Expression levels of Pathogenesis related genes. (**F**) Lesion length (cm). (**G**) Leaf phenotypes of Kitaake and OE-4 plants after inoculation with bacterial blight pathogens. Data are means±SD of three biological replicates (**B**–**E**) and five biological replicates (**F**) (Student’s *t*-test: * *p* < 0.05; ** *p* < 0.01).

To evaluate the biological function of *OsHCAR*, we produced transgenic rice overexpressing the WT *OsHCAR* allele under the control maize (Zea mays) Ubiquitin1 in the Kitaake background. A total of four *OsHCAR* T_0_ overexpression plants (OE) were obtained, and they displayed no apparent morphological changes compared to Kitaake (Figure 5A). The expression levels of *OsHCAR* in the T_0_ OE plants were significantly increased compared to Kitaake (Figure 5B). For further analysis, T1 OE lines were planted in the field, and we measured the activities of SOD and CAT, which are increased during oxidative stress. In SOD activity, the results showed no significant difference between Kitaake and OE lines (Figure 5C). In contrast, CAT activity was significantly higher in OE lines than Kitaake (Figure 5D). 

It has been reported that *HM143* enhances disease resistance to multiple bacterial blight pathogens [9]. To access the disease response to bacterial blight pathogens in the OE lines, we performed bacterial inoculation of five different bacterial blight races, namely C3, C6, JS97-2, PXO347, and PXO349, between the Kitaake and OE-4 lines. We found that the OE-4 lines were more susceptible to bacterial blight pathogens compared to Kitaake, except for PXO347 (Figure 5F,G). We examined the expression pattern of three different pathogenesis-related (PR) genes (*WRKY45*, *WRKY82*, and *WRKY85*). The results showed that these PR genes were poorly expressed in the OE-4 plants compare with Kitaake (Figure 5E). These findings demonstrate that the *OsHCAR* gene is involved in regulating bacterial blight resistance and that overexpression of *OsHCAR* enhances disease susceptibility in rice.

### 2.5. Knockout of OsHCAR Promotes Resistance to Xoo

**Figure 6:**
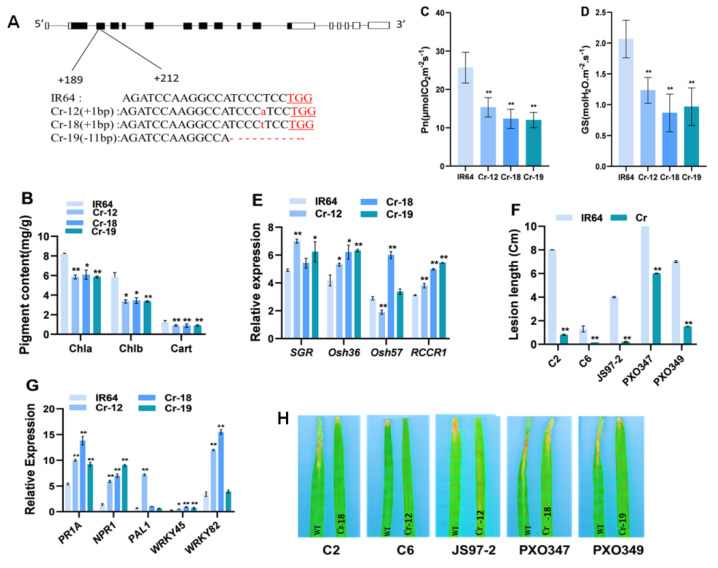
Analysis of *OsHCAR* knockout lines using CRISPR/Cas9. (**A**) CRISPR/CAS9 mediated mutation at target sites of OsHCAR in knockout lines. Cr–12, Cr–18 are homozygous mutants carrying 1 bp insertion whereas Cr-19 carries 11 bp deletion, the black letters represent the sgRNA target sequence and the underlined red letters represents PAM motif. (**B**) Chlorophyll content, (**C**) Net photosynthetic rate (Pn). (**D**) Stomatal conductance (Gs). (**E**) Relative expression of senescence genes. (**F**) Lesion length (cm). (**G**) Relative expression analysis of pathogenesis related genes in WT and knockout lines and (**H**) leaf phenotypes of WT and knockout lines after bacterial blight inoculation. Data are means ± SD of three biological replicates (**B**–**E**,**G**) and five biological replicates (**F**) (Student’s *t*-test: * *p* < 0.05; ** *p* < 0.01).

We used thef CRISPR/Cas9 technique to generate knockout lines of *OsHCAR* using the WT as the background. The sequenced CRISPR/Cas9 target sites revealed several insertions in the cr-12, cr-18, and deletion in the cr-19 (Figure 6A). The three indicative lines were used for comprehensive characterization. To verify the off-target effect, we obtained 20 off-target sites from the CRISPR-P website (http://cbi.hzau.edu.cn/cgi-bin/CRISPR, accessed on 23 June 2021) (Supplementary Table S2), and we detected those off sequence in cr-12, cr-18 and cr-19 by PCR (Polymerase Chain Reaction) amplification and sequencing to verify whether they resulted in any insertion, deletion or substitution mutation. We did not detect any change in cr-12, cr-18, and cr-19 regarding the 20 off-target sites (Supplementary Figures S3–S7), and thus concluded that our guide RNA sequence was only specific to the OsHCAR gene.

As expected, the knockout lines exhibited a spotted leaf phenotype similar to the mutant with decreased levels of Chl a, b, and Cart (Figure 6B) and increased expression levels of senescence-associated genes compared to WT (Figure 6E). We speculated that the reduction in the Chl content in the knockout lines might cause alterations in photosynthetic parameters, so we quantified the photosynthesis parameters. We observed a significant reduction in the *Pn* and stomatal conductance (*GS*) in the knockout lines compared to WT (Figure 6C,D), indicating that the knockout lines had defective photosynthetic capacity, which possibly led to reductions in Chl content and lesion formation.

To analyze the disease resistance, knockout lines and WT plants were inoculated with five races of *Xoo*, i.e., C2, C6, JS97-2, PXO347, and PXO349, and the lesion length was measured two weeks after inoculation. The knockout plants showed enhanced disease resistance to each of the five *Xoo* races compared with WT (Figure 6F,H). PR genes are frequently enhanced during lesion formation in many rice spotted-leaf mutants. During pathogen infection, improved disease resistance is accompanied by the elevation of PR genes [28]. To verify this possibility, we performed an expression analysis of five defense-related marker genes (*PR1A, NPR1, PAL1, WRKY45, WRKY82*) by qRT-PCR. We found that each of these PR marker genes was significantly increased in the knockout plants compared to the WT (Figure 6G). Overall, these results indicate that the dysfunction of *OsHCAR* results in cell death and activates disease resistance in rice.

### 2.6. Constitutively Expressed OsHCAR Is Localized to Chloroplasts

**Figure 7:**
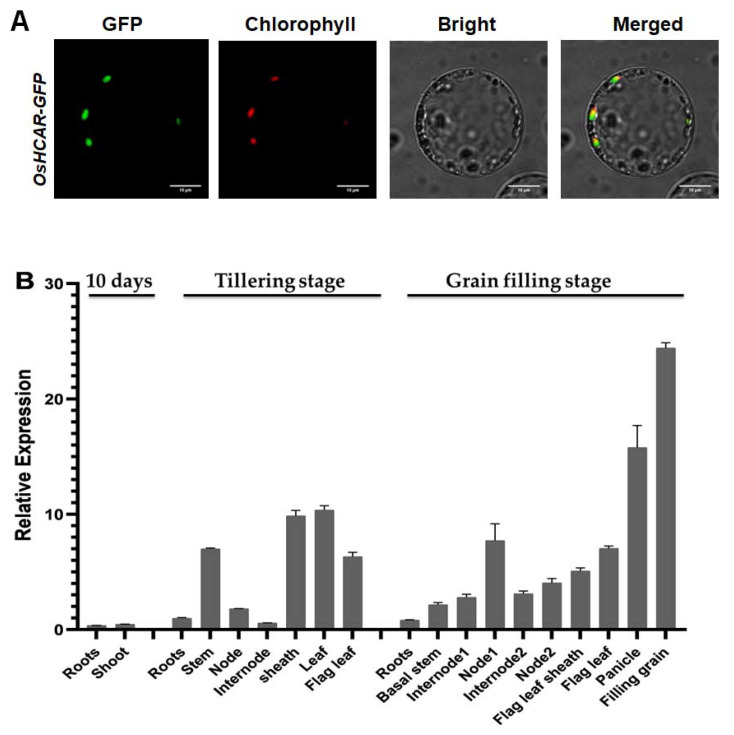
qRT-PCR analysis of wild-type *OsHCAR* messenger RNA expression and subcellular localization of *OsHCAR*. (**A**) Subcellular localization of *OsHCAR-GFP* in rice protoplasts. Bar = 10 µm (**B**) Quantitative relative expression levels of *OsHCAR* in various tissues of IR64 at different growth stages. Rice ubiquitin gene (*LOC_Os03g13170*) was used as reference gene.

To examine the subcellular localization of *OsHCAR* experimentally, we performed polyethylene glycol (PEG)-mediated rice transformation in rice protoplasts. When the pOsHCAR-GFP vector was transformed in rice protoplasts controlled by CaMV 35S promoter, the green fluorescence signals of *OsHCAR: GFP* were confined to the chloroplasts (Figure 7A). Our results demonstrated that *OsHCAR* is a chloroplast-targeted protein. 

To verify the expression system of *OsHCAR*, we evaluated the expression levels of total RNA from roots, shoots, stems, nodes, internodes, leaves, leaf sheaths, panicles, and filling grains by quantitative reverse transcription-polymerase chain reaction at different developmental stages. Our results indicated that the *OsHCAR* gene was highly expressed in all the tissue evaluated, with the highest expression in the filling grains (Figure 7B). These findings reveal that *OsHCAR* is a constitutively expressed gene.

## 3. Discussion

Spotted leaf mutants are essential for comprehending PCD and disease resistance in plants. Previously, we isolated a spotted leaf mutant (*HM143*) from an IR64 treated EMS mutant bank. The target gene was originally termed *spl^HM143^*. *spl^HM143^* exhibited PCD and improved disease resistance to several races of *Xoo* [9]. In the present study, the mutant allele *splHM143,* which possesses one nucleotide replacement at the splicing site, was identified as *LOC_Os04g25400* after fine mapping; and it encodes for a rice 7-hydroxymethyl chlorophyll a reductase (OsHCAR) protein. Complementation analysis using WT *LOC_Os04g25400 could* rescue the spotted leaf phenotype. HCAR catalyzes the conversion of 7-hydroxymethyl Chlorophyll a (7-HMC a) to Chl a; and rice (*Oryza sativa*) genome comprises of one HCAR homolog [24]. *OsHCAR* is highly constitutively expressed and localizes to chloroplasts.

Mutation in many spotted leaf mutants is characterized by the poor performance of essential agronomic traits and decrease in photosynthetic pigments [29]. Still, our results revealed that there was a decrease in photosynthetic pigment. On the contrary, the performance of essential agronomic traits was substantially similar in *HM143* mutant and the knockout lines than WT, suggesting that *OsHACR* may have a potential function in yield improvement due to enhanced disease resistance. *HM143* exhibited a spontaneous leaf lesions resembling HR that occurs after pathogen infection. Previous studies have demonstrated that rice and *Arabidopsis* HCAR proteins regulate cell death and oxidative stress response. The intensity levels of H_2_O_2_ and O^2−^ were much higher in *oshcar* mutant leaves indicated by DAB and NBT staining. Additionally, *JAmyb*, *OsNAC4*, and *OsAPX1* genes that are enhanced during cell death were notably upregulated, suggesting that HCAR plays a vital role in modulating cell death [24]. In this study, cell death in *OsHCAR* was detected using TUNEL assay, an indicator of DNA fragmentation, which was reinforced by the upregulation of *OsMCs* genes which are essential regulators of PCD in plants [26]. H_2_O_2_ is a significant by-product of beta-oxidation and functions as a cue particle to promote cell death. Thus, over-accumulation of H_2_O_2_ is the leading cause of lesion development [30]. Accumulation of MDA causes cellular membrane damage and indirectly affects cell death [31]. Similarly, there was an elevated level of H_2_O_2_ and MDA in *HM143*, indicating an accumulation of cell death which might have contributed to the formation of necrotic lesions. Promoted level of ROS leads to oxidative damage and activation PCD pathway. If ROS appears as a damaging or signaling molecule is determined by the balance between ROS production and scavenging. Detoxing or scavenging of ROS is attained by an effective antioxidative system consisting of enzymatic antioxidants such as SOD, POD, and CAT to balance the production and removal of ROS [32]. Interestingly, CAT, SOD, and POD activities increased significantly in *HM143*; however, the level of ROS accumulation remained high. Further evaluation is needed to elucidate the issue.

In rice and *Arabidopsis*, HCAR participates in preventing cell death signaling during leaf senescence [23]. In general, leaf senescence is unified with reduction of Chl contents, defective chloroplast development, altered expression of photosynthesis-related genes, and elevated levels of Chl biosynthesis genes [27,33,34]. Similarly, in our study, we detected early leaf senescence. Our results revealed a reduction in the content of Chl and upregulation of Chl biosynthesis genes, and lowered expression of photosynthesis-related genes in *HM143*. Besides, we demonstrated decreased Chl content and upregulation of Chl biosynthesis genes in *OsHCAR*-knockout lines, indicating its function in leaf senescence. All the results obtained provided evidence that HCAR-like protein is involved in leaf senescence. It is believed that plant defense responses are regulated by endogenous plant signaling particles, including SA, JA, ethylene, and abscisic acid. For example, the bacterial blight resistance in OsPELOTA has been activated by the salicylic acid metabolic pathway [35], and simultaneously, the enhanced resistance observed in rice *spl40* was due to activation of the SA and JA signaling pathways [19]. The mediated disease resistance of OsSPL24 was incidentally related to numerous pathways/components involved in heat shock proteins, vesicle trafficking, transcription factors, and cell wall components [10]. Salicylic acid (SA) and jasmonic acid (JA) are two practical regulators of defense reaction in plants and are believed to trigger a standard pathogen defense response in rice [36]. This study revealed that expression levels of PR markers involved in SA/JA were obviously elevated in the mutant plants, indicating that this gene is involved in defense response (Supplementary Figure S2A,B). Furthermore, *OsHCAR*-knockout lines improved resistance to rice five races of *Xoo* by inoculation experiments. There was a remarkably increased level of six PR genes in knockdown lines compared to WT. On the contrary, *OsHCAR* overexpressing plants showed susceptibility to five races of *Xoo* tested. Furthermore, the expression levels of three different PR markers were also decreased in the overexpression plants, suggesting that overexpression of *OsHCAR* may lead to the loss of function protein in regulating disease resistance. Previous reports demonstrated the importance of HCAR in Chl disintegration and cell death during leaf senescence [22,24]. This study demonstrated the potential role of *OsHCAR* in controlling bacterial blight pathogens in rice. Currently, transcriptome analysis is being conducted to uncover the molecular mechanism linking the Chlmetabolism pathway and disease resistance.

No previous reports have suggested the essential role of HCAR in defense response in rice. Overall, our results indicated that *splHM143* encoded by HCAR has a significant role in promoting cell death and defense response in rice. 

## 4. Materials and Methods

### 4.1. Plant Materials and Growth Conditions

The WT IR64 and *HM143* mutant obtained from an EMS-induced mutant bank of IR64 were used in this study. During the summer of 2020, the IR64 and *HM143* plants were grown in the paddy field, whereas transgenic rice, including overexpression plants, knockout plants, and complementation plants, were maintained in the greenhouse at the China National Rice Research Institute (CNRRI) in Fuyang, Hangzhou, China during different seasons of the years 2019 and 2020. 

### 4.2. Physico-Biochemical Parameters Measurement

At tillering stage, the pigment contents, such as Chl a, Chl b, and carotenoid (Cart), were evaluated in the top second leaves of *HM143* and IR64 according to the method described by [37]. Similarly, the enzymatic activities of CAT, POD, and (SOD; and the levels of H_2_O_2_ andMDA were determined by employing the respective assay Kit of (Nanjing Jiancheng Bioengineering Institute, Nanjing, China) following the manufacturer’s instructions. The mean value of three biological replicates was employed for analysis by Student’s *t* test, one-way ANOVA and Duncan’s test.

### 4.3. TUNEL Assay

Samples of *HM143* and IR64 were selected at the tillering stage for TUNEL assay following the manufacturer’s requirements of the Fluorescein In Situ Cell Detection kit (Roche, Basel, Switzerland). Fluorescence labelling and sectioning were conducted as previously discussed by [38]. Leaf samples were visualized by viewing with laser scanning confocal microscope (Ceise, Jena, Germany).

### 4.4. Vector Construction

For complementation test, full-length CDS sequence (1800 bp) and promoter sequence (2000 bp) from IR64 were, respectively, amplified and inserted between *HindIII* and *KpnI* sites of pCAMBIA1300 vector. The recombinant vector was transformed into embryogenic calli obtained from *HM143* via agrobacterium-mediated transformation. For overexpression construct, the total length of CDS 1800 bp was amplified from IR64 by PCR and then cloned into pCAMBIA1300 vector powered by maize (Zea mays) Ubiquitin1 promoter. The recombinant vector was transformed into the mature calli generated from Kitaake via *Agrobacterium tumefaciens*-mediated modification. The CRISPR/Cas9 construct for *OsHCAR* was generated according to a previous report [39] and transformed into the calli generated from WT via *Agrobacterium tumefaciens*-mediated modification. All genetic transformations via *Agrobacterium tumefaciens* were performed using the method described by [40].

### 4.5. Quantitative RT-PCR

The total RNA from differing rice tissue, including root, shoot, internode1, node1, internode2, node2, flag leaf sheath, flag leaf, panicle, and filling grain was extracted following to the manufacturer’s instructions using NucleoZOL Reagent Kit (MACHERY-NAGEL, Düren, Germany). Briefly, 0.1 g tissue was homogenized with 1 mL Nucleozol reagent, then 400 µL DEPC (Diethyl Polycarbonate) water was added to the lysate and mixed vigorously for 15 s and incubated for 5–15 min at room temperature. The mixture was centrifuged at 12,000 rpm for 15 min. The supernatant was transferred to a new test tube and 100 mL of 100% isopropanol was added and incubated for 10 min at room temperature. After centrifugation for 10 min at 12,000 rpm, the supernatant was discarded and the pellet was washed twice by adding 500 µL of 75% ethanol and centrifuging for 3 min at 8000 rpm and the total RNA was reconstituted in 20 mL DEPC water. The cDNA was synthesized using the PrimeScript^TM^ RT Master Mix (Perfect Real Time) (Takara, Dalian, China). For the qRT-PCR assay, PowerUp^TM^ SYBR^TM^ Green master Mix kit was employed and conducted on a Thermal Cycler Dice^®^ Real-Time System (Takara, Dalian, China) according to the following steps: Hold: 95 °C 30 s (one cycle); two steps PCR: 95 °C 5 s, 60 °C 30 s (40 cycles); dissociation at 95 °C for 15 s (40 cycles); 60 °C for 30 s, and 95 °C for 15 s. For the reference gene rice ubiquitin (*LOC_Os03g13170)* was utilized. Three replicates were used for all assays and the means were employed for calculations.

### 4.6. Disease Evaluation

At tillering stage, five fully expanded leaves of the WT, knockout lines, Kitaake, and OE-4 plants were inoculated with several bacterial races (C2, C3, C6, JS97-2, PXO347, and PXO349) of *Xoo* at the tillering stage following the leaf-clipping method described by [41]. For inoculation, distilled water was used to suspend bacterial cultures and calibrated to OD_600_ = 1.0. Disease lesion range was calibrated 14 days after inoculation using a ruler and the leaves were photographed by HP Scanjet G4010 scanner machine (HP, Shanghai, China). The mean value of five independent leaves per each bacterial race was used for analysis by Student’s *t* test.

### 4.7. Subcellular Localization

To prepare rice protoplasts, 0.5 g of rice seedlings were sliced into 0.5 mm size and digested in 10 mL enzyme solution (0.6 M Mannitol, 10 mM MES, 1% Cellulose R10, 0.5% Maceroezyme R10, 0.1% BSA, 1 mM CaCl_2_ pH = 7.5) for 6 h in darkness with gentle shaking (60 rpm). The protoplasts were filtered with a nylon mesh and collected by centrifugation, then washed twice with 10 mL ice-cold W5 solution (9% NaCl, 125 mM KCl, 5 mM Glucose, 5 mM MES pH = 5.70) followed by MMG solution (0.4 M mannitol, 15 mM MgCl_2_, 4 mM MES, pH = 5.8), respectively.

The entire coding sequence of *OsHCAR* without a stop codon was amplified from WT (IR64) using specific primers 143CDS-1 and 143CDS-2, respectively (Supplementary Table S1). The PCR product of *OsHCAR* was merged with GFP in PAN580 vector controlled by the CaMV 35S promoter to generate pOsHCAR-GFP construct. The new construct was then co-transiently expressed into rice protoplasts obtained from IR64 seedlings. The GFP signal was detected by viewing using a Zeiss lsm710 confocal microscope (Carl Zeiss, Inc., Jena, Germany) 48 h after transformation.

### 4.8. Map-Based Cloning

Previously, the mutation was mapped to the chromosome 4 [9]. For fine mapping, a total of 1380 F_2_ individuals of mutant-type derived from the cross *HM143*/Moroberekan were used. Simple sequence repeat (SSR) markers were retrieved from the (http://www.gramene.org/, accessed on 23 June 2021) database whereas insertion/deletion (InDel) markers were synthesized using primer 5.0 and DNAStar 8.0 software after sequence comparison between the Indica cultivar 9311 and japonica cultivar Nipponbare from the Gramene public database (http://gramene.org/genome_browser/index.html, accessed on 23 June 2021). Sangon Biotech Co. Ltd. (Shanghai, China) was used to synthesize the primers. PCR reaction and detection were performed as reported before by [8]. The suitable primer sequences for fine mapping are given in Supplementary Table S1.

## Data Availability

Data is contained within the article or supplementary material.

## Supplementary Materials

The following are available online at https://www.mdpi.com/article/10.3390/ijms22147585/s1, Figure S1: Validation of *HM143* by complementation test, Figure S2: Expression analysis of defense genes involved in SA and JA pathways. Table S1: List of primers used in this study, Table S2: List of 20 potential off-target sites, Figures S3–S7: Sequence alignments of off-target sites in three knockout lines (Cr-12, Cr-18, Cr-19).
