# Supplementary material for: A Novel Allele Encoding 7-Hydroxymethyl Chlorophyll a Reductase Confers Bacterial Blight Resistance in Rice
Source: Int J Mol Sci. 2021 Jul 15;22(14):7585. doi: 10.3390/ijms22147585 (PMC8303675; doi:10.3390/ijms22147585)
Supplement: Supplementary file 1 [file ijms-22-07585-s001.zip › ijms-1290083-supplementary.pdf]

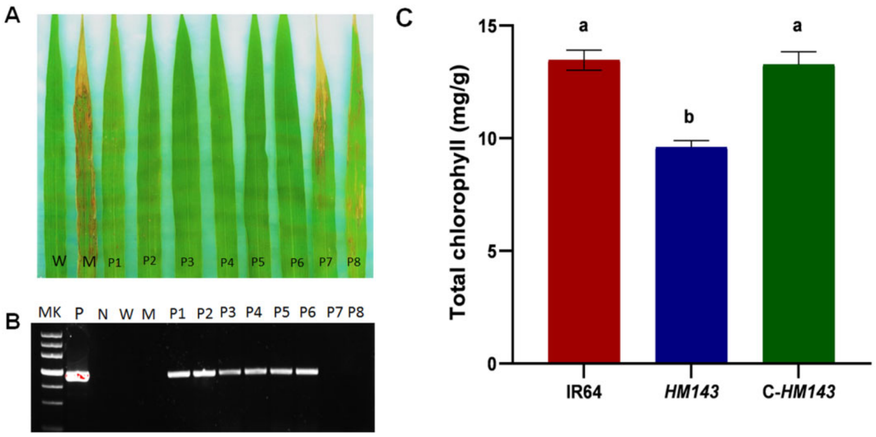

Figure S1. Validation of *HM143* by complementation. **(A)** Leaves of wild type(W), mutant(M), and different complemented plants (P1-P8), (**B)** Positive transformants were identified by detecting the presence of hygromycin marker gene. MK (Marker), P (Positive control), N (No template DNA), W (Wild type), M (Mutant), P1-P8 (different complemented plants). (**C)**Total chlorophyll content in IR64, *HM143* and C-HM143. Different letters indicate significant differences according to One-way ANOVA and Duncan's test ($P \leq 0.05$).

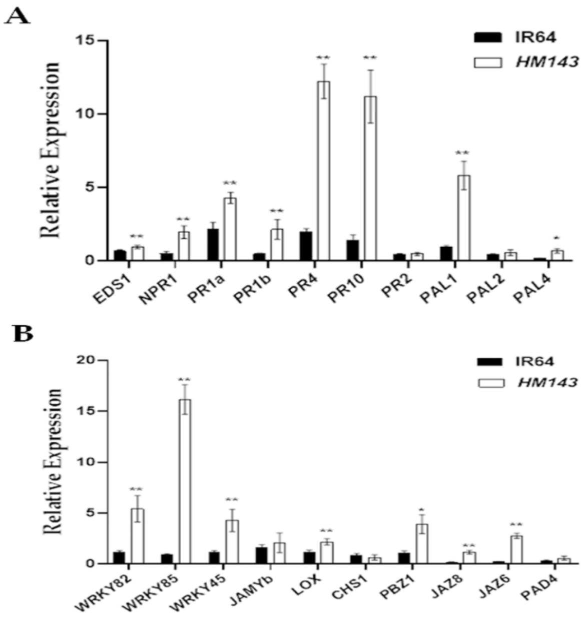

Figure S2. Expression of defense genes involved in SA and JA signaling pathway. **(A)** Expression analysis of SA signaling pathway genes. **(B)** Expression analysis of SA signaling pathway genes. Values are means ± SD of three biological replicates; ** indicates significance at $P \leq 0.01$ and *indicates significance at $P \leq 0.05$ by Student's t test.

Table S1. List of primers used for this study

Primer	Primer sequence (5'-3')	Purpose
143gRNA	AGATCCAAGGCCATCCCTCC	CRISPR/Cas9
123-126-4-F	CACTGGACACGTCTGCAACA	
123-126-4-R	ACGCCTGCACCATCTTGGCCA	
127-130-1-F	GACGCTTAATTGTCAGCACA	Fine mapping
127-130-1-R	TTATGAACAGCTCCTGCCA	
123-126-8-F	GCATTTATAGGATGTCCATG	
123-126-8-R	TAAACAGAGTATGCCAATCC	
126-1-F	ATGTTCTTAATATTTTCTTCC	
126-1-R	CCATGGGACTTGGGAGGTAT	
RM16686-F	GGCACTGCTTGCATATGGATCG	
RM16686-R	TGCCGGCGAACTTATCCTCTCC	
RM16682-F	TGTATTTGCGTGGTGATTGG	
RM16682-R	TTAGGCAGGTACACAATGATGC	
EDS1-F	CATTCCAAGAACGAGGACACTG	
EDS1-R	CAAGACTCAAGGCTAGAACCGA	
NPR1-F	GGCAGGTGAGAGTCTACGAGGAA	
NPR1-R	GCTGTCATCCGAGCTAAGTGTT	
PR1b-F	AGAACTACGCCAGCCAGAGAAG	
PR1b-R	TTCTCGCCAAGGTTGTTCCG	
PR10-F	CACCATCTACACCATGAAGC	
PR10-R	AGCACATCCGACTTTAGGAC	
PAL2-R	GCATCAGCTTCCAACTCG	
PAL2-R	GGTTTCGCACTCCATTACAGA	
PR1a-F	GGAAGTACGGCGAGAACATC	
PR1a-R	TGGTCGATCCACTGCTTCTC	
PR4-F	AGTATGGATGGACCGCCTTCTGT	
PR4-R	CTCGCAATTATTGTCGCACCTGTTC	
PR2-F	GGCAGGTGAGAGTCTACGAGGAA	
PR2-R	GCTGTCATCCGAGCTAAGTGTT	PR genes
PAL4-F	CTTCACAACAGCTAATCGAG	
PAL4-R	CGCACTCCATTTCAGTACCA	
WRKY82-F	AGTGAAAAGTAGTGAAAATTCCAG	
WRKY82-R	GTGCTAGTTTCAATTATTCTGCTTCGT	
WRKY85-F	CAGCAAGAAAAGGAATATACAAAT	
WRKY85-R	CTCAATGTGTTTCCTAACATTACA	
WRKY45-F	TTCCTTGTTGATGTGTCGTCTCA	
WRKY45-R	CCCCCAGCTCATAATCAAGAAC	
JAMYb-F	CCGAGCATGGTGACTAGCTCATCTT	
JAMYb-R	CCTTGCACCCAACCGTTAAGCTGTT	
LOX-F	GATGGCGGTGCTCGACGTGCT	
LOX-R	GCACCTGTTCTTGAGCTTTCTAT	
PBZ1-F	CCCTGCCGAATACGCCTAA	

PBZ1-R	CTCAAACGCCACGAGAATTTG	
CHS1-F	GACTACCCGGACTACTACTTCA	
CHS1-R	CTTCCTGATCGCGACTTG	
JAZ8-F	CCAAACACGGCGGAAACAG	
JAZ8-R	GGTGGACGGGAAGTTCTCAAAG	
JAZ6-F	GGACATGCCGATCGCGAGGAA	
JAZ6-R	GCGCGAGTGCATGTGTCCA	
PAD4-F	CCAACATGTACCGCATCAAG	
PAD4-R	GGTTGTTTCGGTGGTAGTGGC	
OsMC1-F	GCTTCATCAAGGCGGTGGAGT	
OsMC1-R	AAGTTGGCGACCTTGCGGATG	
OsMC2-F	CGACCCGTACAGGGTGCCGA	
OsMC2-R	GCACAGCGCCTCGTCGTAGC	
OsMC3-F	GGCTCCTTCGTCCGCAAGAT	
OsMC3-R	CACAGGAGAAACGGTTTCCTGT	PCD-related
OsMC4-F	TCGACGTTCGTGGAGATGCTC	genes
OsMC4-R	ATTCACGAGCCGCCTGATCTT	
OsMC5-F	GTGCCAGACCGACCAGACAT	
OsMC5-R	CCGCTCTTCTCCGACAGGAT	
OsMC6-F	CCACACCGCAGGGTTCTTCAT	
OsMC6-R	GTCCAGGCTGCTGAGTGTATCC	
OsMC7-F	ATACAGACCGTGCTGGCGTC	
OsMC7-R	AGGAATGGCGTCTCGGCGTT	
OsMC8-F	TCCGGCAAGTGCCTCGTAAC	
OsMC8-R	AGATAACAACGGAAGCATAAAGTC	
RbcL-F	ATCGTGCTCGCGGTATCTTT	
RbcL-R	ACCAGGTGCATTACCCCAAG	
CHLD-F	GGAAAGAGAGGGCATTAG	
CHLD-R	CAATACGATCAAGTAAGTGTT	
cab2R-F	GTTCTCCATGTTCGGCTTCT	
cab2R-R	GACGAAGTTGGTGGCGTAG	
CHLH-F	TGACTCAGACCCGACAAAGC	Photosynthesis-rela
CHLH-R	TCCCCTCGTACCACTTAGGG	ted genes
CHLI-F	CGGAGTAACCTTGGTGCTGT	
CHLI-R	CTTGGCAGCCCTGTTAGTCA	
HEMA1-F	ACACGCCATCTGTTTGAGGT	
HEMA1-R	CAAGCCTCCACTGTTTTGCC	
PsbA-F	TGTAGCTGGTGATTCGGCG	
PsbA-R	ATAACCATGAGCGGCCACAA	
PorA-F	ATCACCAAGGGCTACGTCTC	
PorA-R	GAGTTGTTGTTCCAGCTCCA	
RbcS-F	CCGTGAGAACCACAGATCCC	
RbcS-R	ACGTTGTCGAAGCCGATGAT	
134CDS-1	AGCGTGGGTCTCGGGCTCCATGGCGCGGTGCATCTCCTTCCT	CDS sequence

143CDS-2	AGCGTGGGTCTCGCTGACTGGAGCATCGACTCGATGCGTCCGTCCT	amplification
143-5	GATCACGGTCTCGACCTCCTTGTGTTCGATAC	Promoter sequence
143-6	GCGATAGGTCTCGTGTTTGCACAGTGACCG	amplification
UBI-F	CCCTCCACCTCGTCCTCAG	Reference gene
UBI-R	AGATAACAACGGAAGCATAAAAGTC	
RCCR1-F	CGCATTTCCTCATGGAATTT	
RCCR1-R	CTTCTCACGCTGTTTGTCCA	
Osh36-F	AACGCATTTGTGGTTGGCTC	
Osh36-R	TCAACTTTGGCCGGTGTCTT	Senescence-
Osh57-F	ACCCTAAAGTAAATGAAGTC	Related genes
Osh57-R	CCTGCTCCTTGTCTTGTTAC	
SGR-F	AGGGGTGGTACAACAAGCTG	
SGR-R	GCTCCTTGCGGAAGATGTAG	

Table S2. List of the 20 potential off-target sites

Sequence	Score	Locus	Gene	Region
ACATCCAAGGCCATCCCTCTAAG	8.6	Chr7:-15050489		Intergeniic
AGCTCCAACTCCATCCCTCCTGG	1.4	Chr5:-21075196		Intergenic
GATTCCATGGCCATCCCTCCAGG	1.4	Chr7:+29470914	LOC_Os07g49210	CDS
AGCTCCAAGCCCCTCCCTCCTAG	1.0	Chr7:-10977529		Intergenic
AGCTCCAGCTCCATCCCTCCTGG	0.8	Chr4:+35367918		Intergenic
AGCTCCAGCTCCATCCCTCCTGG	0.8	Chr11:-6133406		Intergenic
AGCTCCAGCTCCATCCCTCCTGG	0.8	Chr8:-17654804		Intergenic
AGCTCCAGCTCCATCCCTCCTGG	0.8	Chr11:+5529310		Intergenic
AGCTCCAGCTCCATCCCTCCTGG	0.8	Chr10:-3948507		Intergenic
AGCTCCAGCTCCATCCCTCCTAG	0.5	Chr9:-17888292		Intergenic
AGCTCCACGTCCATCCATCCGGG	0.5	Chr3:-10215007	LOC_Os03g18210	CDS
AGAATCAAGACCATCCTTCCTAG	0.3	Chr5:+14105545	LOC_Os05g24410	CDS
ACATCCACCGCCATCCCTGCTGG	0.1	Chr8:+9008420		Intergenic
AGCTCCAACTCCACCCCTCCTGG	0.1	Chr1:+6847610		Intergenic
AGCTCCAACTCCACCCCTCCTGG	0.1	Chr8:+11059261	LOC_Os08g18044	Intron
AGCTCCAACTCCACCCCTCCTGG	0.1	Chr5:+19551140		Intergenic
AGCTCCAACTCCACCCCTCCTGG	0.1	Chr11:+3787600	LOC_Os11g07470	Intron
AGCTCCAACTCCACCCCTCCTGG	0.1	Chr3:+26444886		Intergenic
AGCTCCAACTCCACCCCTCCTGG	0.1	Chr4:+24196628		Intergenic
AGCTCCAACTCCACCCCTCCTGG	0.1	Chr12:+6191726		Intergenic

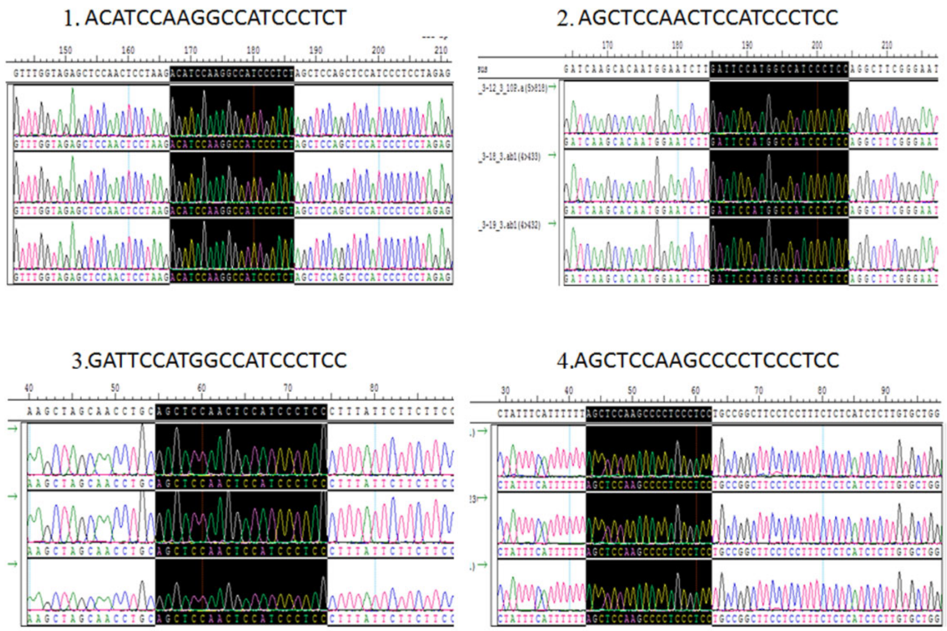

Figure S3. Sequence alignments of off-target sites 1, 2, 3, 4 in Cr-12, Cr-18 and Cr-19 respectively.

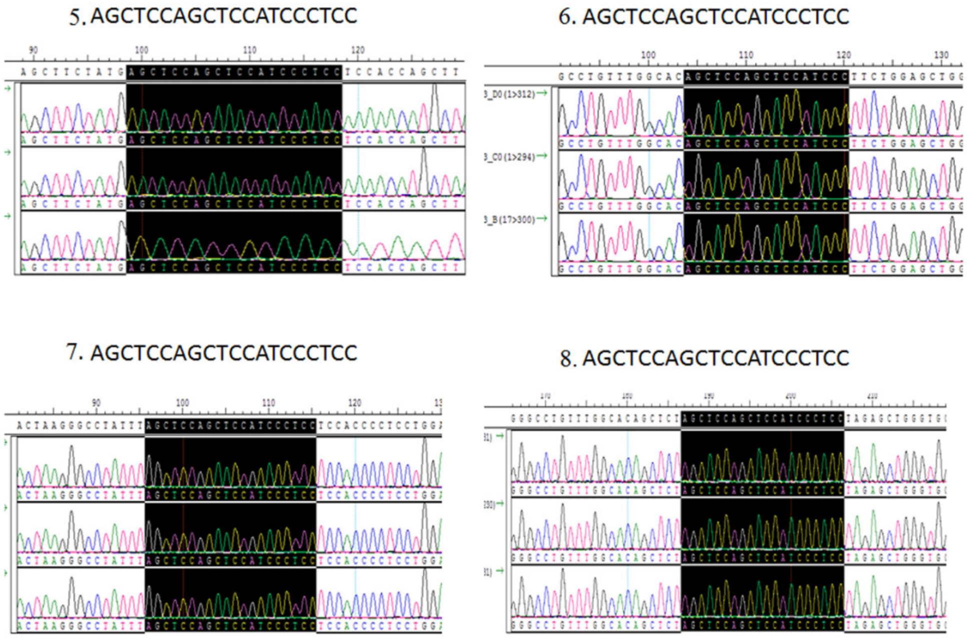

Figure S4. Sequence alignments of off -target sites 5, 6, 7, 8 in Cr-12, Cr-18 and Cr-19 respectively.

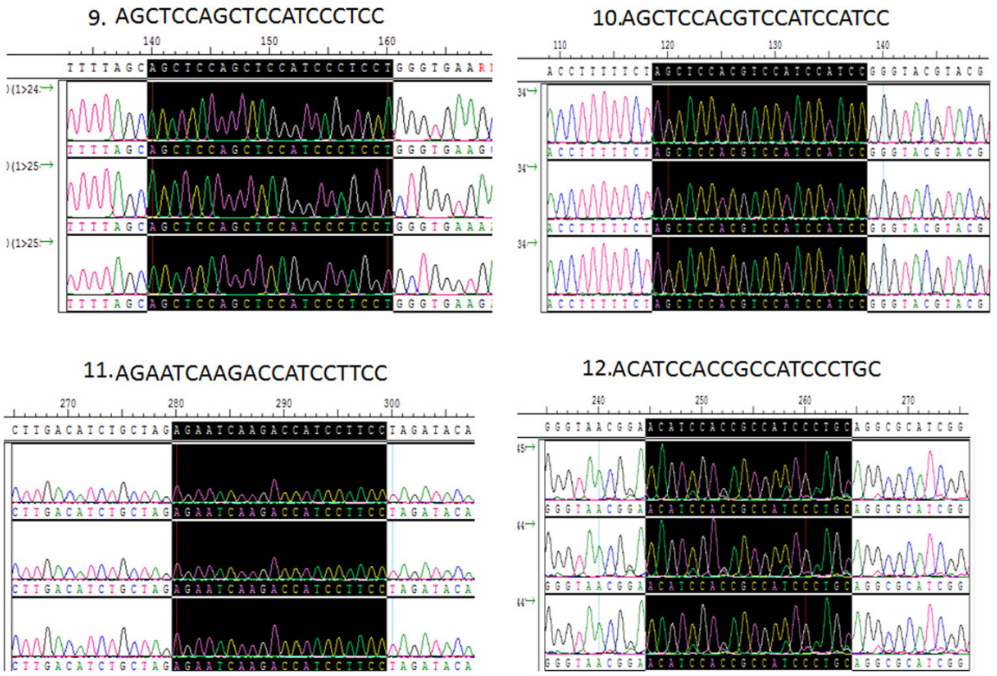

Figure S5. Sequence alignments of off -target sites 9, 10, 11, 12 in Cr-12, Cr-18 and Cr-19 respectively.

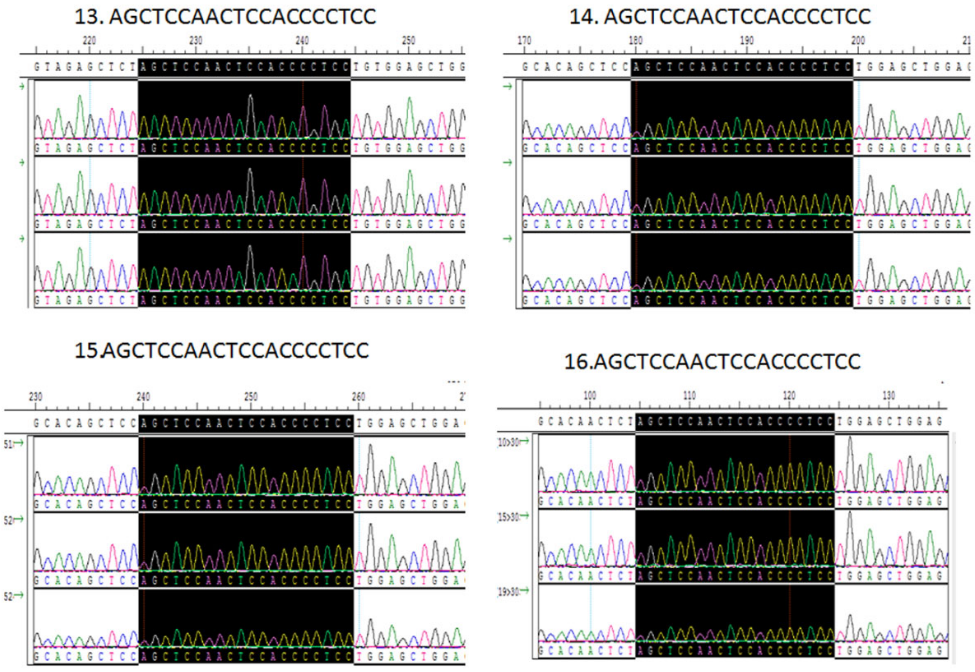

Figure S6. Sequence alignments of off -target sites 13, 14, 15, 16 in Cr-12, Cr-18 and Cr-19 respectively.

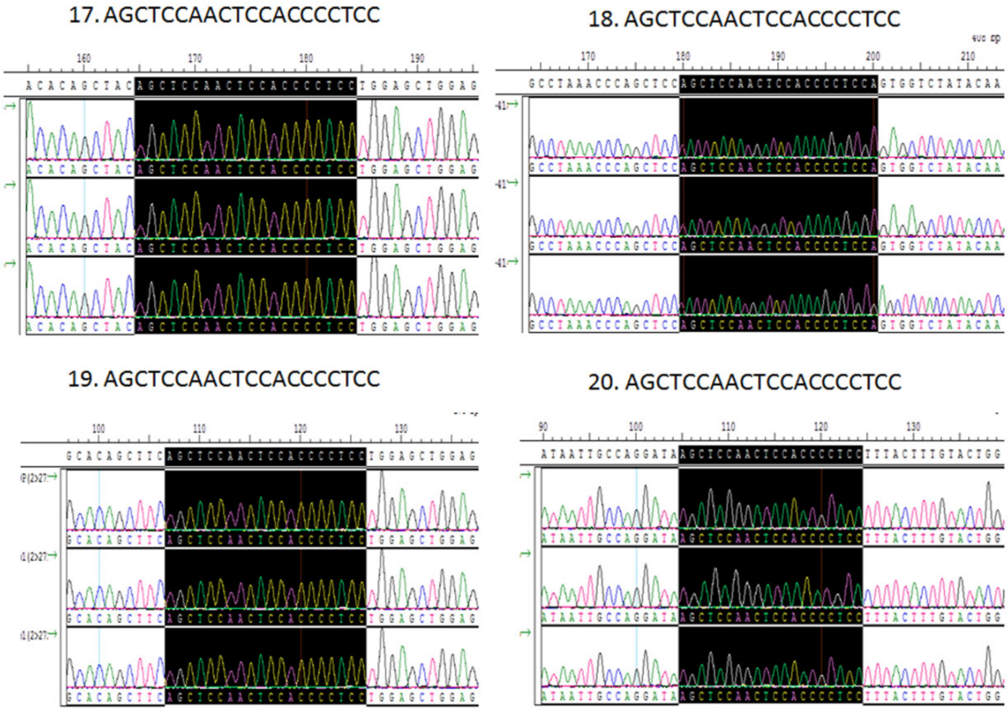

Figure S7. Sequence alignments of off-target sites 17, 18, 19, 20 in Cr-12, Cr-18 and Cr-19 respectively.
